# Intervention Practices for Promoting Well-Being and Cognitive Development in Hospitalized Children: A Scoping Review

**DOI:** 10.3390/ejihpe16030041

**Published:** 2026-03-10

**Authors:** Sofía Castro-Trigo, Alexa von Hagen, Paloma Alonso-Stuyck, Pau Miquel, Donovan Barba-Reynoso, Agustina Quintero, Julieta Zorrilla de San Martín, Augusto Ferreira-Umpiérrez

**Affiliations:** 1 CEU Escuela Internacional de Doctorado (CEINDO), Universidad San Pablo-CEU, CEU Universities, Urbanización Montepríncipe, Av. de Montepríncipe s/n, 28668 Madrid, Spain; 2 Universidad Católica del Uruguay, Av. 8 de Octubre 2738, Montevideo 11600, Uruguay; magustina.quintero@ucu.edu.uy (A.Q.); jzorrilladesanmartin@ucu.edu.uy (J.Z.d.S.M.); auferrei@ucu.edu.uy (A.F.-U.); 3 Independent Researcher, 60316 Frankfurt am Main, Germany; 4 Department of Psychology, Abat Oliba CEU University, CEU Universities, Carrer de Bellesguard 30, 08022 Barcelona, Spain; palonsos895@uao.es; 5 Pediatric Palliative Care and Chronicity Research Group, Sant Joan de Déu Research Institute (IRSJD), Hospital Sant Joan de Déu, Esplugues de Llobregat, 08950 Barcelona, Spain; pau.miquel@sjd.es; 6 School of Pedagogy and Psychology, Universidad Panamericana, Álvaro del Portillo 49, Zapopan 45010, Jalisco, Mexico; dbarba@up.edu.mx

**Keywords:** pediatrics, hospitalization, child development, child health services, cognition, mental health

## Abstract

Psychosocial and cognitive interventions are increasingly implemented in pediatric hospital settings. However, evidence regarding their structure, delivery, and outcomes remains dispersed. This scoping review aimed to synthesize current evidence on these interventions, focusing on their design, professional delivery, reported outcomes, and existing research gaps. It was conducted using established scoping review methodology and is reported in accordance with PRISMA-ScR guidelines. Systematic searches were conducted in PubMed, Scopus, Web of Science, PsycINFO, and ProQuest Dissertations to identify peer-reviewed and grey literature published between 2009 and 2024. Following study selection based on predefined inclusion criteria, data were charted using a standardized data extraction form and analyzed to synthesize and map key characteristics of interventions and outcomes in relation to the review questions. Sixty-one studies met the inclusion criteria. Interventions primarily targeted school-aged children and adolescents and were delivered by psychologists, educators, and nurses, frequently within interdisciplinary teams. A wide range of materials and resources were used, including digital technologies, playful and artistic materials, audiovisual and informational supports, and sensory or therapeutic objects. Techniques primarily involved guided conversation, cognitive and body-based exercises, and play-based approaches. Outcomes mainly focused on emotional well-being and recovery, while fewer interventions explicitly addressed cognitive processes such as attention and executive functioning. Overall, reported effects were generally positive. These findings suggest that psychosocial and cognitive interventions in pediatric hospital settings reflect a wide range of approaches, while also revealing methodological heterogeneity, variability in reporting, and the underrepresentation of low- and middle-income countries, pointing to the need for more robust and inclusive future research.

## 1. Introduction

Advances in medical science and healthcare systems have significantly improved recovery and survival outcomes worldwide. Between 1990 and 2023, the global under-five mortality rate declined by approximately 59%, from 93 to 37 deaths per 1000 live births (World Health Organization, 2025), while life expectancy at birth increased from 66.8 years in 2000 to around 73.3 years in 2024 (Inoue et al., 2019; United Nations, 2024). However, the prevalence of chronic and complex health conditions among children and adolescents remains high, requiring increasingly specific and prolonged medical care (Barrio-Cortés et al., 2020; Leyenaar et al., 2022; National Academies of Sciences, Engineering, and Medicine [NASEM], 2024; Ramachandran et al., 2025; Reif et al., 2022). In this context, pediatric care has gradually evolved from an exclusive focus on disease cure to more holistic and person-centered models that integrate psychosocial well-being and quality of life during and after treatment (Cruz Martín et al., 2018; Pan American Health Organization, 2024; World Health Organization’s Framework on Integrated, People-Centred Health Services, World Health Organization, 2016). Hospitalization represents a unique stressor in the lives of young individuals, potentially leading to a cascade of adverse psychological effects (Cartland et al., 2018).

Hospitalization can disrupt multiple dimensions of child development, extending beyond the physical sphere to impact emotional, social, and cognitive functioning (Fernández de Córdoba & Garay, 2018; Gallegos et al., 2019; García-Salido et al., 2018). Children face disruptions to their daily routines, exposure to painful or unfamiliar medical procedures, and a loss of autonomy, which often provoke uncertainty, dependency, and emotional distress (Ahmed, 2024; Cruz Martín et al., 2018; Lizasoain, 2021). Empirical evidence indicates that hospitalized children are at increased risk of developing symptoms such as anxiety, depression, behavioural disturbances, and low self-esteem (Ahmed, 2024; Barros et al., 2021; Cabral-Gallo et al., 2014). These effects may be especially pronounced among children with chronic health conditions, who represent an estimated 10% to 30% of the pediatric population (Barrio-Cortés et al., 2020; Fardell et al., 2023). Prolonged or repeated hospitalizations have been associated with impaired psychosocial adaptation, delays in developmental milestones, and even signs of post-traumatic stress. In this regard, Meentken et al. (2021) found that 26% of hospitalized children exhibit elevated post-traumatic stress symptoms, with recurrent hospitalizations and parental stress identified as the primary risk factors, consistent with longstanding evidence on psychological adjustment and adaptation to pediatric chronic illness (Wallander & Varni, 1998). Additionally, the hospital setting may foster feelings of social isolation, fear of death, and academic disruption, further intensifying children’s vulnerability (Sillero Sillero et al., 2024; Viotti, 2018). Qualitative studies emphasize the importance of considering children’s subjective perspectives and experiences, revealing nuanced emotional responses and adaptive strategies that are often overlooked in clinical practice (Sillero Sillero et al., 2024). In pediatric research, well-being has been conceptualized as a multidimensional construct encompassing emotional, psychological, social, and functional domains (Pollard & Lee, 2003). Theoretical models further distinguish between hedonic and eudaimonic dimensions of well-being (Ryan & Deci, 2001) and emphasize optimal psychological functioning, adaptation, and purpose as core components of psychological well-being (Ryff, 1989; Ryff & Keyes, 1995). In this review, we therefore focus primarily on emotional and psychological well-being, as reflected in outcomes assessing emotional distress, anxiety, coping, and related indicators, while also capturing selected patient-reported physical well-being indicators where relevant.

School absenteeism and reduced cognitive stimulation are among the main educational challenges associated with pediatric hospitalization. Difficulties have been widely reported in core cognitive domains such as attention, memory, language, reading, writing, and mathematics (Grau, 2005). In a population-based study, Hu et al. (2022) demonstrated that children hospitalized with chronic conditions consistently underperform in reading and numeracy, with a significantly higher risk of failing to meet national academic benchmarks. Children with chronic conditions are 30–40% more likely to present poor academic performance than their healthy peers, and recent studies confirm that prolonged or recurrent hospitalization may further compromise learning and cognitive outcomes, particularly when neurodevelopment is already vulnerable (Fardell et al., 2023). These challenges may persist even after hospital discharge, impacting school reintegration and long-term academic achievement (Spencer et al., 2023). As such, early and integrated cognitive support becomes essential to prevent developmental delays and reduce educational inequalities.

Pediatric hospital-based cognitive and psychosocial interventions are implemented across diverse care contexts, including brief procedural admissions and longer inpatient stays. Accordingly, cognitive-focused interventions may address both short-term procedural demands and broader developmental or educational needs during prolonged hospitalization.

Acknowledging the intricate interplay between health, well-being, and development, healthcare providers are increasingly adopting biopsychosocial approaches to pediatric care. Many pediatric hospitals have incorporated interdisciplinary teams—including educators, psychologists, therapists, child life specialists, and social workers—to design and deliver psychosocial and educational interventions that address the complex needs of hospitalized children. These initiatives aim to support emotional adjustment, procedural coping, and continuity in learning during hospital stays (Fernández de Córdoba & Garay, 2018).

Recent reviews such as those conducted by Abdelkhalik et al. (2024) and Rosenblatt et al. (2024) have highlighted the global expansion of child life services, showing their role in promoting resilience, reducing distress, and enhancing hospital experiences for both patients and families. Some studies have explored the preferences of young patients regarding the design of hospital spaces, emphasizing the importance of elements such as natural light, noise reduction, and a sense of control in mitigating stress and anxiety (Payam et al., 2023). Li et al. (2016) have explored innovative intervention strategies that combine psychosocial support with technological tools or artistic expression. For example, structured play activities have shown effectiveness in reducing anxiety and negative emotions in hospitalized children. Digital interventions, including virtual reality, have been used to reduce pain and distress during medical procedures (Reitze et al., 2024; Ridout et al., 2021), while participatory methods like painting (Abazari et al., 2025) or poetry (Delamerced et al., 2021) have proven useful in accessing children’s emotional experiences during hospitalization. Other studies have reported promising results with immersive technologies for enhancing social connection (Bakker et al., 2018), promoting emotional inclusion and well-being (Thabrew et al., 2022) and using cooperative video games to reduce pre-procedural anxiety and pain (Liszio et al., 2024). Similarly, non-pharmacological approaches such as music therapy have demonstrated efficacy in clinical settings, particularly in reducing pain and anxiety during invasive procedures like lumbar punctures in children with cancer (Nguyen et al., 2010). Some, such as Abdi et al. (2025), Godino et al. (2020) and Sánchez et al. (2024) focus on play, storytelling, laugh-therapy, music, and games to reduce stress and promote expression, while others such as Binarelli et al. (2021) or Olson and Sands (2015) aim to sustain academic activity or support cognitive rehabilitation. Therapeutic environments designed from the patient’s perspective can allow them to understand their preferences, health, disease conditions, and treatment which makes it less traumatic (Cassemiro et al., 2020). Interdisciplinary approaches seem to be some of the most promising approaches, as they allow for a more comprehensive and individualized response to the complex needs of pediatric patients. However, the current body of evidence remains methodologically heterogeneous and lacks standardization in professional roles, delivery formats, and outcome evaluation, limiting the development of robust best practice guidelines.

Given the increasing diversity of approaches, the broad spectrum of professional roles and clinical contexts identified in the literature, a comprehensive scoping review is needed to synthesize the available evidence on current practices. This type of review is particularly relevant in our context, for identifying and mapping key concepts and characteristics found across studies, as well as for reporting and discussing these findings. In contrast to systematic reviews, scoping reviews do not aim to produce a critically appraised and synthesized answer to a specific question, but rather to provide an overview of the existing literature (Zaccagnini & Li, 2023). By consolidating this evidence, we hope to contribute to the development of efficient, coordinated, and evidence-based interventions that support pediatric patients’ full developmental needs within hospital settings. Such efforts can enhance overall well-being and contribute to a more holistic model of pediatric care (Abdi et al., 2025; Sánchez et al., 2024).

### The Present Study

The aim of this study was to systematically identify the available evidence base on interventions focused on promoting the well-being and cognitive development of hospitalized children and adolescents. We sought to identify and summarize past research on the characteristics of the children who attended the interventions, the professionals who implemented the interventions, the interventions focused on promoting well-being and cognitive development, and the impact of these interventions. We also intended to delimit the scope of the available empirical evidence concerning the countries or regions in which studies had been conducted, the languages in which relevant interventions had been developed, and the different research designs or methodologies that had been applied to investigate this topic. More specifically, we aimed to address the following research questions: What is the scope of the available evidence for interventions to promote well-being and cognitive development in hospitalized children and adolescents? What are the characteristics of (a) children and adolescents, (b) professionals and (c) interventions in the available evidence base? What is the impact of the above-mentioned evidence? In Table 1, we detail the PICO components used to define the scoping review.

As recommended by the PRISMA Extension for Scoping Reviews—PRISMA-ScR (Tricco et al., 2018) we formulated broad research questions to guide the review process. The definition of the PICO components was intentionally designed to encompass the diversity of pediatric hospital interventions. This inclusive approach aims to capture a wide array of professional practices and clinical settings, consistent with the mapping objectives of a scoping review (Munn et al., 2018).

## 2. Materials and Methods

Scoping reviews are particularly useful for bringing together literature in disciplines with emerging evidence, as they are well suited to addressing questions beyond those related solely to the effectiveness or experience of an intervention. In addition, scoping reviews may be conducted to determine not only the extent of the research available on a topic, but also how the research has been conducted. To support this broader mapping purpose, scoping reviews usually include a variety of study designs. Accordingly, and in light of the diversity of interventions, settings, and outcomes described in this field, we included multiple study designs in order to comprehensively map the available literature, consistent with established methodological guidance (Peters et al., 2015, 2024). The protocol for this scoping review was preregistered on 30 July 2024 on the Open Science Framework (OSF) and is publicly available at https://doi.org/10.17605/OSF.IO/UHTFX.

### 2.1. Eligibility Criteria

In this review, we planned to only include reports written in Spanish, English, and German, as these are the languages the review team was fluent in. We focused on research published in the last 15 years (2009–2024) and included articles reporting data from qualitative, quantitative, or mixed-methods studies. All types of study designs were included (e.g., cross-sectional, longitudinal, single case studies). Furthermore, we included interventions delivered by health or education professionals, researchers, or students linked to well-being promotion or educational practices in hospital settings.

To be included in the review, studies had to report on the implementation of any kind of intervention to promote well-being and/or cognitive development in hospitalized children and adolescents within hospital settings. To be included, a study had to report on children and/or adolescents up to the age of 18 years as target participants of the intervention. This age cutoff is consistent with international definitions of childhood and paediatric populations, which define children as individuals under 18 years of age (United Nations, 1989, Article 1). Finally, studies needed to report on the effects of the target intervention through at least one of the following outcome measures: perceived and/or evaluated well-being or cognitive outcomes at the individual user (e.g., general well-being, physical well-being), and/or family (e.g., relation, life satisfaction, stress perception) level. Perceived outcomes refer to third-person reports, such as those provided by parents, caregivers, teachers, or other proxies. Evaluated outcomes refer to outcomes based on direct administration of standardized measures or direct clinical observation by a healthcare professional or trained researcher. To facilitate title and abstract as well as full-text screening, we summarized the eligibility criteria for this review in signalling questions (available in the Supplementary Materials, https://osf.io/vr8cx/files/osfstorage, accessed on 10 February 2026).

### 2.2. Search Strategy

The search was conducted on 3 August 2024, using the following databases: ERIC (via EBSCOhost), APA PsycInfo (via EBSCOhost), MEDLINE, Scopus, and Web of Science. Additionally, we searched ProQuest Dissertations to identify grey literature. The search strategy included the following keywords and phrases:

(wellbeing OR cogni* OR educat* OR pedagog* OR teach*)

AND

(intervention* OR program)

AND

(child* OR pediatric*)

AND

(hospital* OR inpatient OR palliative)

For databases that offered language and date filters, we limited the search to reports written in Spanish, English, and German and published from the year 2009 onward. Furthermore, whenever databases allowed for this option, we limited the search to reports that matched the search strategy in their title and/or abstract. Please see Supplementary Materials (https://osf.io/vr8cx/files/osfstorage, accessed on 10 February 2026).

### 2.3. Study Selection Process and Reviewer Workflow

We used the the cloud-based version of Covidence software (Veritas Health Innovation, 2024) to manage our data throughout the review process. As a first step, the lead reviewer conducted database searches and imported full reference details (e.g., titles, abstracts, authors) of all identified records into Covidence. Duplicated entries were automatically detected and removed by the software. Additional duplicates identified during the screening process were manually excluded. Next, two reviewers independently screened the titles and abstracts of all records using Covidence. Any disagreements were resolved through discussion or adjudication by a third reviewer. Records deemed potentially eligible were advanced to full-text screening, and their full-text reports were retrieved and uploaded into Covidence. Finally, two reviewers independently assessed the full-text reports for eligibility, with particular attention to the methods and results sections. Reasons for exclusion at this stage were documented in Covidence. Discrepancies were resolved by a third reviewer’s independent assessment.

Data extraction was performed within Covidence and exported as a .xlsx file. We configured a structured data extraction form based on the following data items: general study characteristics, characteristics of children and adolescents, characteristics of professionals, and characteristics of interventions (see Supplementary Materials, https://osf.io/vr8cx/files/osfstorage, accessed on 10 February 2026). For each included study, one reviewer completed the form, and a second reviewer verified the extracted information by cross-checking it against the original study reports. Discrepancies were discussed and resolved by consensus. To synthesize the evidence we identified, we computed frequencies and percentages for each data item and complement this information with examples of included studies, using the SWiM reporting guideline from Campbell et al. (2020).

*AI-assisted language editing*

During the preparation of this manuscript, an AI-based tool (Grammarly) was used exclusively to assist with language improvement and proofreading (e.g., grammar, clarity, and style). The tool was not used to generate any scientific content, data, analyses, or interpretations.

## 3. Results

**Figure 1:**
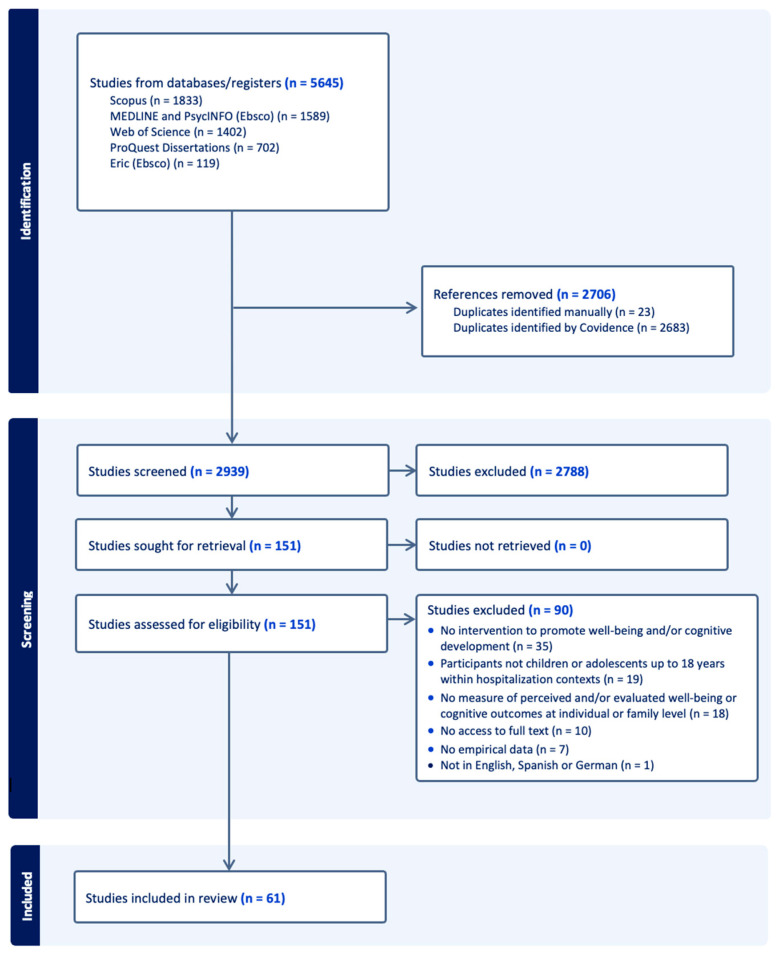
PRISMA Flow Chart.

In Figure 1 we depict the results of our study selection process using the PRISMA flow chart (Tricco et al., 2018). After deduplication, the search results consisted of 2939 records that underwent title and abstract screening. Next, we assessed 151 studies at the full-text screening stage, of which 61 met inclusion criteria for this scoping review. The complete tables of the 61 included studies and the 90 excluded studies, including reasons for exclusion, are available on the Open Science Framework (OSF).

The results presented below in aggregated form across the 61 included studies are organized according to key characteristics of participants, interventions, professional involvement, and reported outcomes. Given the scope of the review, full study-level details are provided in the publicly available OSF dataset (https://osf.io/vr8cx/files/osfstorage, accessed on 10 February 2026), which allows readers to examine and filter the extracted data by variables such as age group, clinical context, and study design.

### 3.1. The Scope of the Available Evidence on Interventions to Promote Well-Being and Cognitive Development in Hospitalized Children and Adolescents

#### 3.1.1. Publication Year

**Figure 2:**
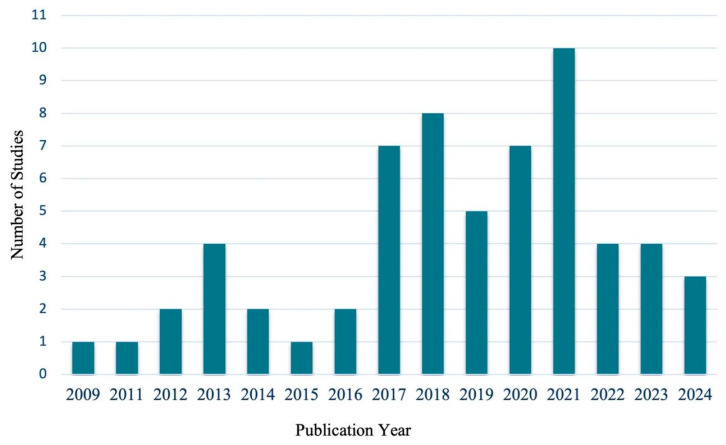
Number of Studies by Year of Publication (*n* = 61).

The distribution of the included studies across publication years is shown in Figure 2. This overview illustrates a shift in the volume of publications over time. Prior to 2017, an average of approximately two studies per year were published, whereas from 2017 onward the average increased to approximately six studies per year. While publication volume peaked in 2021, the overall period since 2017 reflects a higher sustained level of research activity compared to earlier years.

#### 3.1.2. Geographical Distribution

**Figure 3:**
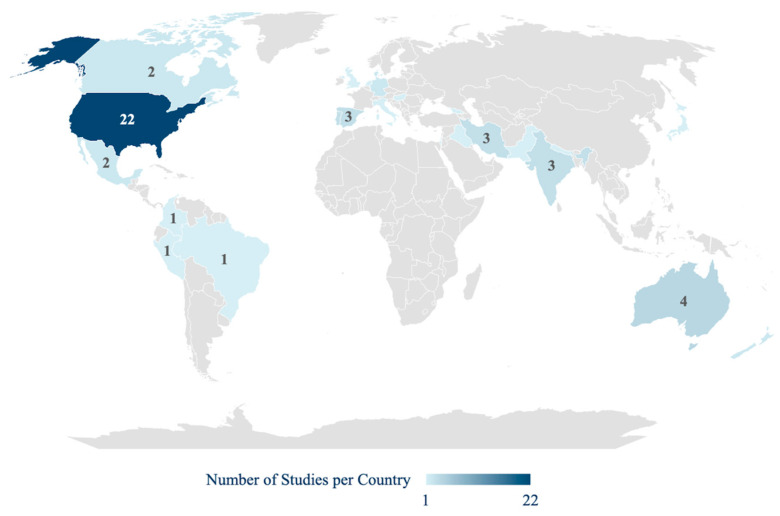
Geographical Distribution of Included Studies by Country (*n* = 61).

Most of the studies were conducted in the United States (*n* = 22). Other countries represented were Australia (*n* = 4), India (*n* = 3), Spain (*n* = 3), Iran (*n* = 3), Portugal (*n* = 3), Canada (*n* = 2), New Zealand (*n* = 2), Denmark (*n* = 2), Germany (*n* = 2), and Mexico (*n* = 2). Additionally, Peru, Georgia, Hungary, Israel, Nepal, Brazil, Japan, Netherlands, United Kingdom, Iraq, Colombia, Italy, and Pakistan were each represented by one study (Figure 3).

#### 3.1.3. Publication Type and Study Design

In total, 50 studies were published in peer-reviewed journals, while 11 were categorized as grey literature. The most frequent approach was the use of quantitative experimental designs (*n* = 18; 29.5%), which were commonly conducted in the context of specific, short-term intervention instances. Of the 18 quantitative studies included, ten were randomized controlled trials (RCTs). Among the RCTs, interventions ranged from multi-component group-based adapted CBT for children and adolescents (Kilburn et al., 2023) to brief, procedure-focused interventions using the TICK-B coloring book and coloring materials during cannulation (Suleman et al., 2022). The remaining 8 used non-randomized quantitative designs. For instance, Zamani et al. (2022) implemented caregiver-led play stimulation sessions using age-appropriate materials, resulting in significant improvements in patients’ mental status. Similarly, Caruso et al. (2021) employed portable VR headsets and the Space Pups™ application, which significantly increased positive affect and patient cooperation while reducing anxiety during clinical procedures. Shifting from intervention-based research, the review identified 16 studies (26.2%) that utilized quantitative non-experimental designs, such as cross-sectional, longitudinal, or observational studies. This methodological approach was particularly prevalent in research spanning the entire hospital stay, as seen in the work of Stadler et al. (2024), Gilbey et al. (2023), and Zucchetti et al. (2020). Twelve studies (19.7%) employed more than one methodological category, typically using mixed methods. In contrast, nine studies (14.8%) were strictly based on qualitative designs. For instance, Bakker et al. (2018) explored the experiences of patients using live streaming with virtual reality, while Villadsen et al. (2015) conducted a qualitative investigation into the impact of a social-pedagogical intervention on young patients facing long-term hospital admissions. Finally, six studies (9.8%) utilized single-case designs or other atypical methodological frameworks. A notable example is the work of Dolidze et al. (2013), who presented a clinical model of Child Life intervention specifically designed to facilitate effective coping strategies in a pediatric patient undergoing heart surgery.

#### 3.1.4. Participants and Data Sources

Data were most frequently collected from more than one participant group per study. Overall, 60 studies (98.3%) reported data obtained from children or adolescents, while in 28 studies (45.9%) parents were also represented. In total, 8 studies (13.1%) each included a broader category of caregivers and other participants such as teachers, hospital staff, and volunteers. Only one study (1.6%) included siblings.

#### 3.1.5. Intervention

In terms of intervention format, individual interventions were the most common, described in 49 studies (66.2%). Group-based formats appeared in 24 studies (32.4%). In addition, four studies (5.4%) included hybrid arrangements combining individual and group sessions, family-centered formats in which participants engaged jointly, or unstructured digital environments with asynchronous interaction. Only one study (1.4%) did not provide clear information regarding how participants were organized during the intervention.

#### 3.1.6. Outcomes

Outcomes related to the individual well-being of children or adolescents were the most frequently assessed, appearing in 53 studies (86.9%). These outcomes were primarily measured through self-report questionnaires, observational tools, or standardized assessments completed by the children or adolescents themselves, targeting emotional, behavioural, or psychosocial processes. In addition, some studies included external ratings provided by clinicians or researchers. In 24 studies (39.3%), family members served as informants, providing data through questionnaires, interviews, or observational reports. In most of these cases, parents reported on children’s emotional state, behaviour, or perceived response to the intervention. Cognitive outcomes at the individual level were assessed in 20 studies (32.8%), using tools such as memory tasks, attention assessments, or academic performance indicators. Only three studies (4.9%) involved cognitive data reported by or about family members, such as parental beliefs or understanding of children’s cognitive development.

### 3.2. Characteristics of the Children and Adolescents Involved in Interventions

#### 3.2.1. Demographic Description of the Pediatric Population

For analytical clarity, information on participants’ age was grouped into three developmental stages: early childhood (0–5 years), school age (6–12 years), and adolescence (13–18 years). School-aged children were the most frequently included group, appearing in 51 studies (83.6%), followed by adolescents in 39 studies (63.9%), and young children in 18 studies (29.5%). Several studies included participants from more than one age group, accounting for totals exceeding the number of included studies. With respect to sex, the majority of studies (*n* = 50, 82.0%) included both male and female participants, while a smaller number focused exclusively on males (*n* = 4, 6.6%) or females (*n* = 2, 3.3%). In five studies (8.2%), no information on sex was provided. Socioeconomic status (SES) was the least frequently reported participant characteristic: 42 studies (68.9%) did not report any SES-related information. Among the remaining studies, most described samples with heterogeneous socioeconomic backgrounds (*n* = 15, 24.6%), while only a small number explicitly focused on specific SES groups, including low SES populations (e.g., Bharuchi & Rasheed, 2024) or moderate-to-high SES samples (e.g., Stadler et al., 2024). Studies conducted across different countries and healthcare systems varied considerably in whether and how SES was operationalized. Lack of SES reporting was most frequently observed in studies conducted in high-income countries. By contrast, several studies from low- and middle-income countries explicitly described participants’ socioeconomic background or targeted socially disadvantaged groups.

#### 3.2.2. Participants’ Hospitalization Context

**Figure 4:**
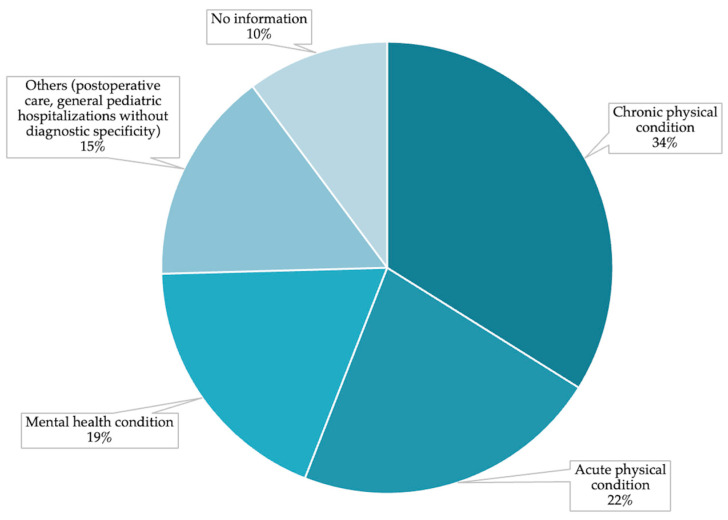
Percentage of Hospitalized Children and Adolescents by Medical Condition.

Across the reviewed studies, chronic physical conditions were the most frequently reported health issues addressed by interventions, appearing in 20 studies (32.8%). Acute physical conditions were reported in 13 studies (21.3%), while mental health disorders were the focus in 11 studies (18.0%). Nine studies (14.8%) were classified as “others” which included postoperative recovery, general pediatric hospitalizations without diagnostic specificity, and palliative care situations. In six studies (9.8%), no information regarding participants’ medical conditions was provided (Figure 4).

Regarding the type of care setting, acute hospitalization was the most commonly reported context described in 22 studies (36.1%, e.g., Sil et al., 2021; Suleman et al., 2022), followed by medium and long-stay hospitalizations in 21 studies (34.4%, e.g., Stadler et al., 2024; Zucchetti et al., 2020). Ambulatory care settings were reported in 10 studies (16.4%), while 12 studies (19.7%, e.g., Gilbey et al., 2023; Kilburn et al., 2023) did not specify the type of hospitalization.

Information on the length of hospital stays varied across studies with no information in 29 cases (47.5%). Among the remaining studies, 16 (26.2%) involved hospitalizations of more than 15 days, predominantly associated with mental health diseases and chronic physical conditions, 8 (13.1%) described stays of three to seven days, mostly involving children with acute physical conditions, and 5 (8.2%) reported hospitalizations of more than seven but fewer than 15 days, related to both chronic and mental health conditions. Shorter stays, lasting one to two days, were mentioned in three studies and were exclusively linked to acute physical conditions or specific medical procedures (4.9%).

As for previous hospitalization experience, 41 studies (67.2%) did not report this information. Of the remaining studies, 17 (27.9%) involved participants with prior hospitalization experience, and only three studies (4.9%) included participants hospitalized for the first time.

### 3.3. Characteristics of the Professionals Delivering the Interventions

#### 3.3.1. Professional Profiles Involved in the Interventions

A wide range of professional profiles were involved in delivering the interventions. Among the most frequently reported were psychologists (*n* = 24), educators (*n* = 18), nurses (*n* = 15), occupational therapists (*n* = 13), doctors (*n* = 11), and social workers (*n* = 6). In addition, educational psychologists, school psychologists and/or special needs educators were identified in four studies, and caregivers or volunteers participated in another four. Only five studies (8.2%) did not report who implemented the intervention. In total, 30 studies (49.2%) included other details which revealed the involvement of interdisciplinary teams, including combinations of psychologists, educators, creative therapists, art and play specialists, and, in some cases, researchers or supervised students.

#### 3.3.2. Professional Experience and Training

Regarding professional experience and training, 56 studies (91.8%) did not report whether implementers had prior experience in hospital settings. Only four studies (6.6%) specified that the intervention was delivered by professionals with more than five years of experience, and one study (1.6%) reported implementers with one to three years of hospital-based experience. Concerning the background and training of those responsible for delivering the interventions, most studies (*n* = 38, 62.3%) identified practitioners—such as psychologists, educators, or specialized therapists—as the main implementers. In 13 studies (21.3%), the interventions were carried out by researchers, while only six studies (9.8%) did not specify the professional identity of the implementers. A small number of studies (*n* = 2, 3.3%) involved students as implementers. Two additional studies described either collaborative efforts between students and senior practitioners or the participation of non-professional facilitators with specific training. In terms of familiarity with the intervention, 36 studies (59.0%) reported that implementers had received prior training in the methodology or technique used, five studies (8.2%) explicitly noted the absence of such training, and 19 studies (31.1%) did not provide this information.

### 3.4. Characteristics and Components of the Interventions Implemented with Hospitalized Children and Adolescents

#### 3.4.1. Duration and Frequency

In terms of how the intervention was scheduled over the course of hospitalization, 38 studies (62.3%) described the intervention as occurring during specific instances (e.g., targeted sessions, procedural moments, or scheduled activities). In total, 16 studies (26.2%) reported that the intervention spanned the entire hospital stay of the child or adolescent. Another four studies (6.6%) provided no information. In addition, three studies (4.9%) referred to interventions offered optionally, depending on the day of the week or the availability of the professional in charge. Studies involving chronic physical conditions and mental health conditions were more often conducted during medium and long inpatient stays, whereas studies focusing on acute physical conditions were more frequently conducted in acute hospitalization or short-term care contexts. Concerning the frequency with which interventions were delivered, 19 studies (31.1%) reported implementation every day, while seven studies (11.5%) delivered the intervention more than twice a week. Another three studies (4.9%) implemented interventions twice a week, and two (3.3%) once a week. A substantial number (14 studies, 23.0%) provided no information on frequency. Additionally, 15 studies (24.6%) were categorized as Other, which included variable or flexible schedules depending on patient needs, availability of professionals, or contextual conditions such as short stays or shared responsibilities among teams.

#### 3.4.2. Group Size and Intervention Setting

Regarding the size of the group in which interventions were delivered, the vast majority of studies (47 studies, 77.0%) reported interventions conducted in a one-on-one format. Another 22 studies (36.1%) described interventions delivered in small groups of three to ten participants. Additionally, three studies (4.9%) used a pair-based format, and another three studies (4.9%) reported large group formats with more than 10 participants. Only four studies (6.6%) provided no information on group size. Some studies reported using more than one format, so categories are not mutually exclusive. As for the settings where interventions took place, the most frequently reported was the hospital individual room, with 26 studies (42.6%) using this space. Day hospitals and shared hospital rooms were each mentioned in 12 studies (19.7%). Interventions conducted in the hospital school were reported in four studies (6.6%). The category “other” was present in nine studies (14.8%) and included diverse settings such as playrooms, therapy-specific rooms, corridors or waiting areas, and outdoor spaces. In 15 studies (24.6%) the specific location was not reported.

#### 3.4.3. Treatment Integrity

With regard to the use of intervention manuals, 17 studies (27.9%) explicitly reported the use of a structured manual to guide the implementation of the intervention. In contrast, 21 studies (34.4%) stated that no manual was used in the development or delivery of the intervention. A considerable number of studies—22 out of 61 (36.1%)—provided no information on whether an intervention manual was used.

#### 3.4.4. Intervention Materials and Resources

**Figure 5:**
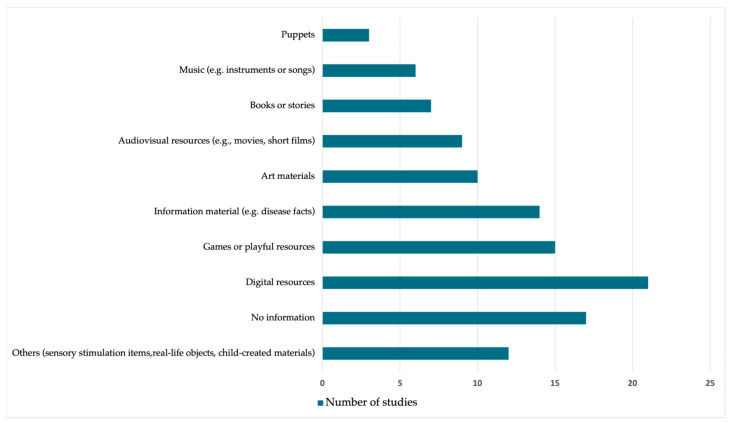
Type of Materials and Resources Used in the Interventions.

A wide variety of materials and resources were employed across the interventions reviewed (Figure 5), which can be broadly classified into categories such as digital technologies, artistic tools, informative resources, sensory and interactive elements, and therapeutic play objects. Digital resources (e.g., apps, tablets, virtual reality) were reported in 21 studies (34.4%) (e.g., Flujas-Contreras et al., 2019; Thabrew et al., 2022), while games or playful resources appeared in 15 studies (24.6%) (e.g., Bharuchi & Rasheed, 2024; Logan et al., 2019). Information materials such as brochures or factsheets were included in 14 studies (23.0%) (e.g., Major et al., 2017), and art materials (e.g., drawing, painting supplies) in ten studies (16.4%) (e.g., Zucchetti et al., 2020). Audiovisual resources (e.g., short films, animations) were mentioned in nine studies (14.8%), and books or stories in seven studies (11.5%) (e.g., Kilburn et al., 2023). Music-based tools were employed in six studies (9.8%) (e.g., Chivington, 2016), and puppets in three studies (4.9%). In 12 studies (19.7%), the materials were categorized as “others”, which included sensory stimulation items (e.g., textures, soft objects), projection mapping for immersive environments, real-life objects (e.g., stethoscopes, hospital tools) used for therapeutic familiarization, and child-created materials (e.g., emotion charts or progress maps). Overall, 17 studies (27.9%) provided no specific information on materials or resources used.

#### 3.4.5. Intervention Strategies and Techniques

**Figure 6:**
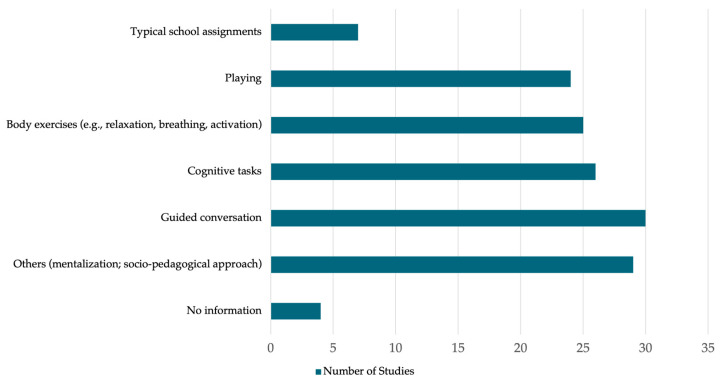
Strategies and Techniques Used in the Interventions.

The techniques employed in the interventions are closely related to the materials and resources described above, yet they represent distinct components of implementation. While materials refer to the tools or media used, techniques reflect how the specific modes or strategies through which those resources are delivered and experienced. Interventions utilized a broad range of techniques (Figure 6). Interventions employed a diverse range of techniques (Figure 6), distinct from the materials used. The most frequent were guided conversation (49.2%), including clinical interviews and structured dialogues (e.g., Fennig et al., 2017; Gilbey et al., 2023), and cognitive tasks (42.6%) such as restructuring and neuropsychological rehabilitation (e.g., Goldstein et al., 2018; Kilburn et al., 2023). Body-based exercises (41.0%), including relaxation and physical activation, were used for stress management and motor recovery (e.g., Chivington, 2016; Lagarde, 2018; Stadler et al., 2024). Play-based techniques (39.3%) spanned from therapeutic play to social robotics (e.g., Bharuchi & Rasheed, 2024; Logan et al., 2019), while school assignments (11.5%) supported educational continuity (e.g., Bastidas-Rivera et al., 2023; Goldstein et al., 2018; McClure, 2017). Notably, 47.5% of studies utilized other techniques. These included sensory and expressive-arts interventions (e.g., drawing, music, aromatherapy) for emotional externalization (Chivington, 2016; Pottinger, 2017; Zucchetti et al., 2020), and trauma-focused or mentalization-based strategies for emotional regulation (Cabrera et al., 2020; Gilbey et al., 2023; Toyama, 2021). Additionally, socio-pedagogical strategies such as visual supports and social stories were used for neurodiversity (Kilburn et al., 2023) or early childhood pedagogical intervention (Zucchetti et al., 2020). Finally, relational methods like Goal Attainment Scaling (GAS) (Stadler et al., 2024) and virtual telepresence (Thabrew et al., 2022) were used to sustain social connections and functional goals.

#### 3.4.6. Cognitive Domains

**Figure 7:**
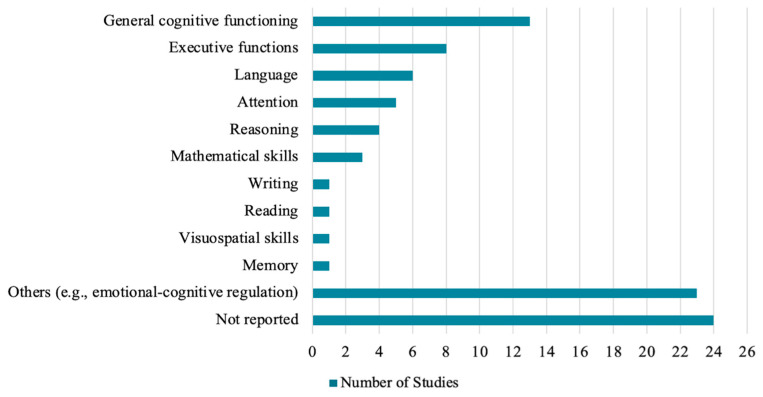
Cognitive Domains Targeted in Interventions.

A wide range of cognitive processes were addressed in the reviewed interventions, though 24 studies (39.3%) did not report this information explicitly. General cognitive functioning was the most targeted domain, mentioned in 13 studies (21.3%) (e.g., Bharuchi & Rasheed, 2024; Zucchetti et al., 2020). More specific processes were less frequently described: executive functions were addressed in eight studies (13.1%) (e.g., Goldstein et al., 2018; Kilburn et al., 2023), language skills in six studies (9.8%) (e.g., Zucchetti et al., 2020), attention in five studies (8.2%) (e.g., Goldstein et al., 2018), and reasoning in four studies (6.6%) (e.g., Kilburn et al., 2023). Mathematical skills were targeted in three studies (4.9%) (e.g., Goldstein et al., 2018), and memory, visuospatial skills, reading, and writing were each addressed in one study (1.6%) (e.g., Goldstein et al., 2018). In 23 studies (37.7%), interventions were classified under “Others”, focusing on emotional–cognitive regulation (e.g., Cabrera et al., 2020; Gilbey et al., 2023), general problem-solving strategies (e.g., Major et al., 2017), metacognitive awareness, and social cognition (e.g., Logan et al., 2019). These were often embedded in broader psychoeducational approaches or in creative and play-based activities, rather than being isolated cognitive training tasks (Figure 7).

#### 3.4.7. Well-Being Domains

**Figure 8:**
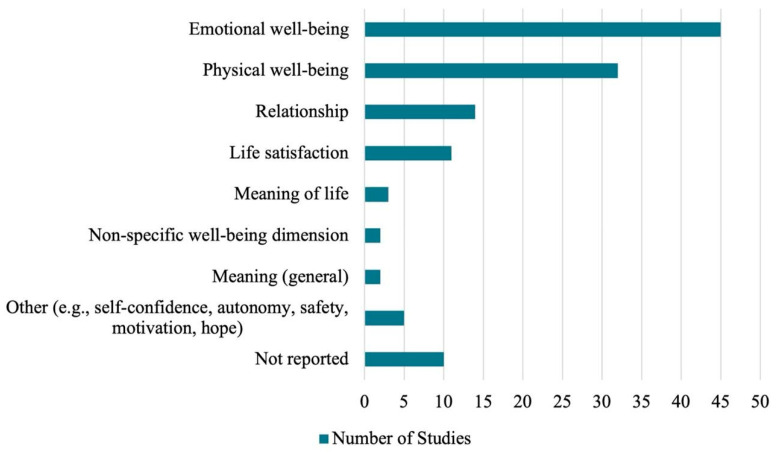
Well-being Dimensions Targeted in Interventions.

The interventions reviewed addressed a variety of well-being dimensions (Figure 8), with emotional well-being appearing as the most targeted, featured in 45 studies (73.8%) (e.g., Cabrera et al., 2020; Gilbey et al., 2023; Hitchcock et al., 2022; Kilburn et al., 2023). Physical well-being was addressed in 32 studies (52.5%) (e.g., Chivington, 2016; Stadler et al., 2024; Taguchi et al., 2018), often in connection with symptom management or activity-based recovery. Relationship-related well-being was considered in 14 studies (23.0%) (e.g., Logan et al., 2019; Major et al., 2017; Thabrew et al., 2022), including peer, sibling, and caregiver-child dynamics. Life satisfaction was included in 11 studies (18.0%) (e.g., Flujas-Contreras et al., 2019). Some studies explored more existential or abstract dimensions, such as meaning of life (three studies, 4.9%) and meaning in general (two studies, 3.3%). Additionally, two studies (3.3%) referred to non-specific well-being, without clear delineation of the dimension. Overall, five studies (8.2%) included other items such as self-confidence, autonomy (e.g., Taguchi et al., 2018), sense of safety, motivation, and hope (e.g., Zucchetti et al., 2020). Notably, studies conducted across the entire hospital stay more often reported multiple wellbeing dimensions, including emotional and physical wellbeing, as in Stadler et al. (2024) and Gilbey et al. (2023). Another ten studies (16.4%) did not report information on the well-being dimensions they aimed to influence.

### 3.5. Reported Impact of the Interventions

Across the reviewed studies, the impact of psychosocial, cognitive, and educational interventions on hospitalized children and adolescents was reported in diverse ways, reflecting both qualitative and quantitative approaches. In 53 studies (86.9%), outcomes related to individual well-being were perceived or evaluated, including improvements in emotional expression, anxiety reduction, engagement, and adaptability to the hospital environment. For instance, organized play sessions were found to significantly lower behavioral distress scores (Bhama et al., 2018), while the use of live-streaming virtual reality allowed patients to maintain social connections, effectively reducing boredom and the stress of separation from home (Bakker et al., 2018). In 24 studies (39.3%), outcomes extended to the family level, highlighting reduced caregiver stress, enhanced communication, and improved family dynamics during hospitalization. Technological platforms such as the GetWellNetwork demonstrated this by significantly increasing “Family Informed” satisfaction scores, improving how parents perceived the quality of education and care provided (Kompany et al., 2016). Regarding cognitive impact, 20 studies (32.8%) addressed perceived or evaluated changes at the child or adolescent level, most commonly referring to improvements in attention, memory, problem-solving skills, or school engagement. Specifically, interventions involving educational robotics and “unplugged” programming fostered computational thinking and collaborative problem-solving (González-González et al., 2021). Other studies using specialized music pedagogy reported that the use of customized technology helped children achieve “flow experiences,” which increased their educational stamina and motivation to learn despite their medical condition (Issaka & Hopkins, 2017). However, only three studies (4.9%) explored cognitive outcomes in the context of parental perception of children’s learning capacity or adaptation. It is important to note that the type of outcome reported varied significantly across studies. Some relied on structured instruments and pre-post designs, such as the use of Goal Attainment Scaling (GAS) to measure functional gains in neuropediatric rehabilitation (Stadler et al., 2024), while others used observational data or participant self-reports. Despite this variability, the vast majority of interventions were associated with positive outcomes, and no study explicitly reported negative effects. A minority of studies noted neutral or inconclusive results, particularly in pilot interventions or those still in preliminary stages of implementation, or where physical recovery (e.g., BMI) did not immediately translate into changes in core cognitive perceptions (Fennig et al., 2017).

## 4. Discussion

The findings of this scoping review provide a comprehensive overview of the current landscape of interventions aimed at promoting well-being and cognitive development in hospitalized children and adolescents. When examined in terms of average publication volume, the temporal distribution indicates a higher mean number of studies per year from 2017 onward compared with earlier years. Recent studies (e.g., Toyama, 2021; Alade, 2021) exemplify the continued application of psychological and cognitive strategies in pediatric care, but overall the data suggest fluctuating research activity with a modest increase in average output in the later period rather than a sustained upward trend.

Geographically, the evidence base is notably concentrated in high-income countries, with the United States accounting for 29.5% of the evidence base (*n* = 18) (e.g., Chivington, 2016; Pottinger, 2017). This predominance may reflect stronger research infrastructure and funding availability in these settings. However, the inclusion of studies from diverse regions, such as Noblecilla Castro (2019) in Latin America and Dolidze et al. (2013) in Georgia, highlights a growing global interest in this domain. The scarcity of evidence from low- and middle-income countries signals a critical gap and an area where further research is needed.

While the review shows a clear predominance of quantitative approaches—particularly experimental and observational designs emphasizing measurable outcomes through validated clinical and developmental instruments such as the WISC-IV for cognitive assessment (Goldstein et al., 2018) or Goal Attainment Scaling (GAS) for functional progress (Stadler et al., 2024)—it also includes a significant number of qualitative and mixed-methods studies (e.g., Duda, 2013; Pottinger, 2017) that contribute valuable insights into the contextual and experiential dimensions of interventions. In addition, single-case studies, small-sample designs, and other exploratory or non-standard methodologies (e.g., Toyama, 2021) offer promising avenues for future research. These approaches are particularly well suited to explore how interventions function within specific populations or health conditions, and could help address more nuanced questions around individualization, adaptability, and contextual fit in pediatric hospital settings.

The reviewed studies predominantly targeted school-aged children and adolescents, while early childhood was less frequently addressed. This distribution may be explained by the increased developmental readiness for structured interventions among older children and the methodological challenges of conducting interventions with younger populations. For example, Lee (2019) and Pottinger (2017) implemented psychosocial strategies in hospital settings specifically designed for school-aged participants, while Flujas-Contreras et al. (2019) included both children and adolescents in their therapeutic protocols.

Although socioeconomic status (SES) was seldom reported, its consideration is essential for assessing the equity and contextual relevance of interventions. A few studies explicitly included children from socioeconomically vulnerable settings—such as Allah Yar and Kazemi (2020), which focused on under-resourced educational contexts in Iran—highlighting the need for culturally and contextually sensitive approaches to psychosocial support. In most cases, participants’ SES was either absent or described in general terms, revealing heterogeneous and poorly characterized backgrounds. This limitation restricts the capacity to understand how interventions may function across diverse social contexts. Consistently defining and reporting SES in future research is a valuable recommendation, as it would enhance the ability to adapt and tailor interventions to the specific needs of different populations. In this regard, incorporating a culture-sensitive perspective into psychosocial care is equally important. Considering the cultural context of the child—including language, customs, beliefs, and spirituality—can contribute to more effective interventions that are aligned with the lived experiences and concrete needs of each population, as noted by Cassemiro et al. (2020). Furthermore, our findings reveal that the impact of these interventions transcends the patient, improving family dynamics. Technological integration and clear communication pathways have shown to increase ‘Family Informed’ scores, suggesting that reducing parental uncertainty may be a component of successful pediatric psychosocial care (Kompany et al., 2016).

Given the high and growing prevalence of chronic and complex health conditions among children and adolescents, more than half of the interventions (*n* = 32; 52.5%) were specifically designed for populations with long-term medical needs. This trend reflects a clinical reality in which sustained psychosocial and cognitive support becomes essential throughout extended treatment trajectories. For instance, interventions targeting pediatric cancer (Abedini et al., 2021; Arriaga et al., 2020; González-González et al., 2019; Taguchi et al., 2018), chronic pain (Major et al., 2017; Toyama, 2021), diabetes (Berger et al., 2017), and palliative care contexts (Duda, 2013) illustrate this orientation toward chronic illness. In contrast, a smaller group of studies (*n* = 5; 8.2%) focused on acute conditions, such as children hospitalized for heart surgery (Dolidze et al., 2013) or traumatic brain injury (Alade, 2021), where the intervention addressed short-term but intensive medical episodes. A distinct group of interventions was developed for children and adolescents in psychiatric inpatient units (*n* = 10; 16.4%), reflecting medical conditions rooted in mental health, including severe psychiatric disorders (Bobier et al., 2009), anorexia nervosa (Fennig et al., 2017), suicide risk management (Esposito-Smythers et al., 2011) and inpatient psychiatric care more broadly (Cabrera et al., 2020). Additionally, several studies targeted general pediatric populations without specifying a particular diagnosis, thus broadening the applicability of the interventions (González-González et al., 2021; Lee, 2019; Muñoz-Arteaga et al., 2021; Ranamagar & Karki, 2021). Despite this clinical heterogeneity, many studies did not report on the duration or frequency of hospitalizations, limiting the ability to draw conclusions about how these variables may influence intervention outcomes. Interventions targeting cognitive and developmental domains were more commonly implemented in populations with chronic or long-term conditions, where repeated or prolonged hospitalizations are expected (e.g., Goldstein et al., 2018; Zucchetti et al., 2020). In contrast, studies in acute care contexts more frequently focused on emotional distress and procedural coping (e.g., Bharuchi & Rasheed, 2024; Chivington, 2016). However, inconsistent reporting of hospitalization duration limited more detailed analyses.

This diversity of approaches is partly attributable to the broad inclusion criteria inherent to the scoping review methodology, and partly reflects the complexity and multidisciplinary nature of interventions targeting hospitalized children’s well-being and cognitive development. In this field, interventions necessarily span different clinical contexts (e.g., child psychiatry in Kilburn et al., 2023; oncology in Zucchetti et al., 2020; and physical rehabilitation in Taguchi et al., 2018), professional roles, and developmental targets, which contributes to a heterogeneous evidence base.

Regarding the professional profile of those delivering the interventions, the analysis revealed the involvement of a wide range of professionals in the implementation of psychosocial and cognitive interventions. Most frequently, psychologists, nurses, educators, and therapists delivered the interventions, either individually or as part of interdisciplinary teams. Indeed, psychologists played a central role (*n* = 18; 29.5%), as reflected in interventions by Fennig et al. (2017) and Toyama (2021). For example, Duda (2013) described the role of music therapists in facilitating emotional expression and encourage bonding and legacy building, while Issaka and Hopkins (2017) highlighted the role of music teachers in promoting learning and socialization. Other interventions emphasize the collaboration between educators and medical staff to support hospitalized children’s school continuity (Dunlap et al., 2013; Ellis et al., 2013). Moreover, in many cases, interventions are delivered by both educators and occupational therapists such as those by Alade (2021) and Potasz et al. (2013). Psychologists also play a key role, as reflected in many of the reviewed interventions (e.g., Fennig et al., 2017; Gómez Zarco et al., 2018; Toyama, 2021), and the role of volunteers and/or caregivers emerges as a relevant factor in several included studies (Logan et al., 2019; Noblecilla Castro, 2019; Pottinger, 2017). The collaborative engagement of different professionals in these interventions reflects an evolving understanding of healthcare that acknowledges the significance of addressing both the physiological and psychological dimensions of illness, potentially leading to more comprehensive and effective treatment paradigms.

Although the majority of interventions were delivered by trained professionals, only one study (1.6%) (Kilburn et al., 2023) offered detailed descriptions of the training processes or hospital experience of the implementers. This omission presents a challenge in evaluating the consistency and fidelity of the interventions. In some cases, such as Kilburn et al. (2023) the study clearly described both the intervention and the training in the Cognitive Behavioural Therapy (CBT) program. Supervised student involvement was reported in a small number of cases. However, the lack of detailed supervision protocols, qualifications of facilitators, or interdisciplinary coordination in many studies suggests a need for improved reporting standards to ensure replicability and intervention quality. Documenting the competencies and backgrounds of intervention facilitators is crucial for assessing the real-world feasibility and scalability of these interventions in diverse hospital settings.

Overall, the heterogeneity in professional roles and the limited reporting on training and implementation highlight key areas for improvement in the literature. It seems reasonable to expect that, beyond professional titles, the knowledge and training of implementers in developmental processes may be relevant for understanding how interventions are delivered and interpreted. In the reviewed studies, implementers ranged from specialized clinical psychologists and multidisciplinary teams (e.g., Kilburn et al., 2023; Stadler et al., 2024) to educators (e.g., Zucchetti et al., 2020) and even social robots acting as mediators (e.g., Logan et al., 2019). However, based on the current state of the evidence identified in this scoping review, there is insufficient and inconsistent reporting to draw firm conclusions regarding how implementers’ training relates to intervention delivery or outcomes.

With respect to the characteristics of the interventions and their impact, the interventions reviewed demonstrated high variability in delivery formats and content. Most were individualized, although some incorporated group-based formats that promoted peer interaction and normalized hospital experiences, as seen in the work by Pottinger (2017). Common strategies included guided conversation, cognitive exercises, and play-based activities. Digital tools such as tablets, animations, and educational apps were increasingly used to facilitate engagement and learning, exemplified by Lee (2019) and Noblecilla Castro (2019). Virtual reality also emerged as a prominent feature in some studies such as Thabrew et al. (2022) and Bakker et al. (2018), highlighting its potential to enhance immersion and emotional involvement in hospital-based interventions. Art-based (e.g., Ranamagar & Karki, 2021; Suleman et al., 2022) and music-based techniques (e.g., Chivington, 2016) also featured, aiming to support self-expression and emotional regulation. Body relaxation and breathing exercises were incorporated into multiple interventions, as demonstrated by Cabrera et al. (2020) and Kilburn et al. (2023) to reduce distress and anxiety and to enhance emotional well-being. While hospital rooms remained the primary setting, interventions were also implemented in hospital schools and day hospitals, indicating spatial flexibility.

Consistent with the broad inclusion criteria of this scoping review, substantial variation in intervention formats and content was expected. This variety of formats, techniques, and settings illustrates the adaptability of psychosocial and cognitive interventions to the dynamic and multifaceted nature of pediatric hospitalization. Rather than relying on a one-size-fits-all approach, many interventions are purposefully designed to respond to the specific emotional, cognitive, and contextual needs of children and adolescents. In this sense, diversity is not just inevitable but necessary for responsive and developmentally appropriate care. Future research should focus on how to optimize this flexibility, recognizing that effective interventions need not be complex. Instead of aiming for an ideal model, efforts should prioritize feasible, context-sensitive strategies that can make a meaningful impact with the resources available.

However, the broad scope of our findings indicates that the primary challenge for the field is not a lack of interventions, but a lack of conceptual and terminological consistency. This suggests that the current state of the literature may benefit more from the development of unified frameworks than from the mere proliferation of new programs. Such frameworks would allow for a more cohesive comparison between studies, bridging the gaps between different clinical settings and the diverse professional backgrounds of implementers.

Overall, findings reported reductions in anxiety, increased engagement, and improvements in emotional states across settings. However, follow-up assessments were scarce, and long-term effects remain underexplored. Furthermore, cognitive domains such as general functioning, executive functions, and language skills were occasionally addressed in interventions described by Alade (2021), Bobier et al. (2009) and Caruso et al. (2021). Academic skills, including mathematics and literacy, are more frequently addressed in interventions framed within school support programs (Goldstein et al., 2018; Muñoz-Arteaga et al., 2021; Noblecilla Castro, 2019). Emotional and physical well-being were the most targeted outcomes (e.g., Dolidze et al., 2013; Pottinger, 2017; Ranamagar & Karki, 2021) although some studies also explored social and relational aspects (Duda, 2013; Dunlap et al., 2013). This uneven focus on emotional over cognitive outcomes raises important considerations for future research and practice. Given the potential developmental impact of prolonged hospitalization, limited cognitive stimulation during this period may have lasting effects on executive functioning, language development, and learning readiness. Moreover, the term cognitive encompasses a broad and heterogeneous set of processes, which are often insufficiently defined in the reviewed studies. Differentiating between core cognitive functions—such as attention, memory, reasoning, and language— and academic skills like reading, writing, or math could help clarify intervention goals and mechanisms of change. Defining the specific domains or abilities targeted is therefore essential not only for evaluating impact, but also for designing developmentally appropriate and contextually relevant programs. This aligns with existing evidence on the educational and developmental risks associated with pediatric hospitalization, including school absenteeism, cognitive delays, and long-term academic underperformance (Fardell et al., 2023; Hu et al., 2022). Addressing these challenges requires integrated and preventive cognitive support within biopsychosocial frameworks (Fernández de Córdoba & Garay, 2018) reinforcing the need to broaden intervention targets beyond emotional adjustment, and to systematically include cognitive development as a core component of comprehensive pediatric care. A concrete example of how these dimensions can be conjugated in practice is illustrated by the hospital early childcare model described by Zucchetti et al. (2020). In their intervention, cognitive stimulation focusing on language, logic, and coordination is not treated as a separate task but is delivered within a framework of emotional support facilitated by psycho-oncologists and educators. This synergy suggests that emotional stability and cognitive engagement are interdependent processes; the secure professional relationship provides the necessary foundation for the child to participate in developmental tasks, while the successful completion of these tasks reinforces the child’s sense of self-efficacy and emotional well-being. Such models exemplify that the integration of both domains is achievable through multidisciplinary collaboration, treating the child’s development as a unified experience rather than a set of isolated variables.

### Limitations

The findings of this scoping review should be interpreted as descriptive and mapping-oriented, providing a solid foundation for understanding the landscape of pediatric hospital interventions. By mapping the reporting of implementation variables—such as professional training and socioeconomic factors—this study establishes a baseline that may support the future development of more standardized reporting practices, ultimately facilitating the scalability and replicability of interventions across diverse hospital settings. To support this process, the comprehensive tables in the Supplementary Materials allow users to filter the evidence by key categories—such as intervention type, age group, or medical condition—enabling a customized search tailored to specific clinical or research needs.

A further consideration for future research relates to the refinement of the search strategy. Given the variability of terminology across disciplines and clinical contexts, some relevant studies employing alternative terms may not have been captured. While the terms selected aligned with the descriptive mapping aims and ensured a feasible retrieval across multiple databases, future reviews could further optimize sensitivity. In particular, the outcome-related block could be complemented with more specific terms—such as anxiety, distress, pain, or coping—to capture studies that focus on these dimensions without explicitly using broader well-being terminology. Similarly, the intervention-related block could be broadened to include descriptors such as “therapy,” “treatment,” or “support service.” An example of an expanded search strategy is provided in the Supplementary Materials to guide future investigators in building upon this work. Accordingly, the findings should be interpreted within the conceptual boundaries defined for this review, which guided both the formulation of the research questions and the operationalization of the search strategy.

Furthermore, as this review reflects the evidence available at the time of the search, the rapidly evolving nature of pediatric psychosocial care suggests that periodic updates will be important to capture emerging technological and clinical innovations.

Consistent with the aims of a scoping review, the broad inclusion strategy was designed to map the breadth of existing practices and provide a comprehensive overview of the field. However, this breadth serves as a necessary first step rather than a final basis for Evidence-Based Practice (EBP). At this stage, the evidence is sufficient to provide a comprehensive landscape, but not to dictate specific clinical guidelines. Future research should now leverage this mapping to embark on systematic reviews (SRs) that target specific diagnostic groups or hospitalization profiles, providing the depth required to evaluate efficacy and inform tailored clinical pathways.

## 5. Conclusions

This scoping review maps a heterogeneous landscape of interventions for hospitalized children’s emotional and cognitive development. The included studies describe a wide range of approaches, delivery formats, and professional profiles, reflecting the diversity of practices currently reported in the literature.

For the daily hospital routine, this review offers a valuable resource for psychologists, educators, and multidisciplinary teams by illustrating how cognitive stimulation and emotional support can coexist within medical treatment. This mapping is grounded in a person-centered model of care, highlighting the diverse range of existing tools, from digital and virtual reality applications to art-based and psychoeducational support strategies, that are currently used to address child development in a more comprehensive manner. This approach acknowledges that children’s experience of illness and recovery is simultaneously emotional, cognitive, and physical. By identifying approaches where these dimensions are successfully conjugated, this review highlights the potential for integrated support where emotional well-being and cognitive stimulation act as mutually reinforcing catalysts for children’s overall development and recovery.

Nonetheless, several gaps persist. This mapping identifies a marked scarcity of data regarding socioeconomic status, implementer training, and long-term follow-up, limiting the ability to assess the equity and replicability of the evidence base. For the research community, documenting these omissions establishes a baseline that identifies where further work can be developed. Future research can build upon this mapping to embark on systematic reviews that target specific diagnostic groups, allowing for a more focused evaluation of efficacy. Ultimately, this review establishes a foundation for strengthening multidisciplinary collaboration, ensuring that cognitive development and emotional well-being are symmetrically addressed throughout children’s hospitalization trajectory.

## Figures and Tables

**Table 1:** PICO Components.

PICO Component	Delimitation
Participants	hospitalized children and adolescents
Intervention	all kinds of interventions on well-being and cognitive development in hospitalized children and adolescents
Comparison	not applicable
Outcomes	perceived and/or evaluated outcomes at individual user, and/or family level.

## Data Availability

The data supporting the findings of this study are available in the Open Science Framework (OSF) repository at https://osf.io/vr8cx/ (accessed on 10 February 2026). This repository includes the extracted data and Supplementary Materials used in the scoping review.

## Supplementary Materials

The following supporting information can be downloaded at https://osf.io/vr8cx/files/osfstorage (accessed on 10 February 2026): Excluded Studies; Final Dataset of Included Studies; Signaling Questions; Full Search Strategy; Search Strings; Data Extraction Form; Suggestions for Expanded Database Search Strategies.

## References

[B1-ejihpe-16-00041] Abazari L., Ghonchehpour A., Abazari A., Isari Z., Abbaszadeh M. H., Tavan A. (2025). Experiences of children during hospitalization: Content analysis of interviews and paintings. BMC Pediatrics.

[B2-ejihpe-16-00041] Abdelkhalik M., Ibrahim M., Al Maaz F., Boutros E. (2024). Integrating child life specialists in pediatric oncology and hematology care: A narrative review. The Journal of Pediatric Academy.

[B3-ejihpe-16-00041] Abdi F., Karamoozian A., Lotfilou M., Gholami F., Shaterian N., Niasar A. A., Aghapour E., Jandaghian-Bidgoli M. (2025). Effect of play therapy and storytelling on the anxiety level of hospitalized children: A randomized controlled trial. BMC Complementary Medicine and Therapies.

[B4-ejihpe-16-00041] Abedini S., Habibi M., Abedini N., Achenbach T., Semple R. (2021). A randomized clinical trial of a modified mindfulness-based cognitive therapy for children hospitalized with cancer. Mindfulness.

[B5-ejihpe-16-00041] Ahmed E. A. (2024). Psychological impact of hospitalization on child and family and the role of nursing care. International Journal of Psychology Sciences.

[B6-ejihpe-16-00041] Alade K. (2021). Examination of cognitive flexibility over time in response to a cognitive rehabilitation program in pediatric traumatic brain injury patients.

[B7-ejihpe-16-00041] Allah Yar M., Kazemi F. (2020). The role of dish gardens on the physical and neuropsychological improvement of hospitalized children. Urban Forestry & Urban Greening.

[B8-ejihpe-16-00041] Arriaga P., Melo A. S., Caires S. (2020). The effects of hospital clowning on physical and emotional states of pediatric patients during chemotherapy treatment. Child & Youth Care Forum.

[B9-ejihpe-16-00041] Bakker A., Janssen L., Noordam C. (2018). Home to hospital live streaming with virtual reality goggles: A qualitative study exploring the experiences of hospitalized children. JMIR Pediatrics and Parenting.

[B10-ejihpe-16-00041] Barrio-Cortés J., Fernández C. S., de Oliveira M. B., Lagos C. M., Beca-Martínez M. T., Lozano-Hernández C., del Cura-González I. (2020). Chronic diseases in the paediatric population: Comorbidities and use of primary care services. Anales de Pediatría (English Edition).

[B11-ejihpe-16-00041] Barros I., Lourenço M., Nunes E., Charepe Z. (2021). Intervenções de enfermagem promotoras da adaptação da criança/jovem/família à hospitalização: Uma scoping review. Enfermería Global.

[B12-ejihpe-16-00041] Bastidas-Rivera S., Sánchez J. E., Sierra Y., González L. (2023). The hospital classroom, an opportunity for educational inclusion, PREPRINT (Version 1). Research Square.

[B13-ejihpe-16-00041] Berger B., Sethe D., Hilgard D., Martin D., Heusser P. (2017). Design of a self-management program for children aged 6–12 years with type 1 diabetes mellitus at the community hospital herdecke, Germany. Complementary Medicine Research.

[B14-ejihpe-16-00041] Bhama O. M., Anila K. P., Philip T. A. (2018). Effectiveness of an organized play session among hospitalized children and parents’ attitude. Indian Journal of Public Health Research & Development.

[B15-ejihpe-16-00041] Bharuchi V. N. A., Rasheed M. A. (2024). Effect of play-based intervention on children’s mental status and caregiver involvement during hospitalization: Findings from Pakistan. BMC Pediatrics.

[B16-ejihpe-16-00041] Binarelli G., Joly F., Tron L., Lefevre Arbogast S., Lange M. (2021). Management of cancer-related cognitive impairment: A systematic review of computerized cognitive stimulation and computerized physical activity. Cancers.

[B17-ejihpe-16-00041] Bobier C., Dowell J., Swadi H. (2009). An examination of frequent nursing interventions and outcomes in an adolescent psychiatric inpatient unit. International Journal of Mental Health Nursing.

[B18-ejihpe-16-00041] Cabral-Gallo M. d. C., Delgadillo-Hernández A. O., Flores-Herrera E. M., Sánchez-Zubieta F. A. (2014). Manejo de la ansiedad en el paciente pediátrico oncológico y su cuidador durante la hospitalización a través de musicoterapia. Psicooncología.

[B19-ejihpe-16-00041] Cabrera N., Moffitt G., Jairam R., Barton G. (2020). An intensive form of trauma focused cognitive behaviour therapy in an acute adolescent inpatient unit: An uncontrolled open trial. Clinical Child Psychology and Psychiatry.

[B20-ejihpe-16-00041] Campbell M., McKenzie J. E., Sowden A., Katikireddi S. V., Brennan S. E., Ellis S., Hartmann-Boyce J., Ryan R., Shepperd S., Thomas J., Welch V., Thomson H. (2020). Synthesis without meta-analysis (SWiM) in systematic reviews: Reporting guideline. BMJ.

[B21-ejihpe-16-00041] Cartland J., Ruch-Ross H. S., Carr L. E., Hall A., Olsen R., Rosendale E., Ruohonen S. (2018). The role of hospital design in reducing anxiety for pediatric patients. HERD Health Environments Research & Design Journal.

[B22-ejihpe-16-00041] Caruso T. J., Fonseca A., Barreau A., Khoury M., Menendez M., Wang E., Lawrence K., Jackson C., Rodriguez S. (2021). Real time reorientation and cognitive load adjustment allow for broad application of virtual reality in a pediatric hospital. Journal of Clinical and Translational Research.

[B23-ejihpe-16-00041] Cassemiro L. K. D. d. S., Okido A. C. C., Furtado M. C. d. C., de Lima R. A. G. (2020). The hospital designed by hospitalized children and adolescents. Revista Brasileira de Enfermagem.

[B24-ejihpe-16-00041] Chivington K. J. (2016). The effects of music therapy and harmonica with pediatric patients admitted for respiratory issues. Master’s thesis.

[B25-ejihpe-16-00041] Cruz Martín O., Hernández Meléndez D. E., Pérez Inerárity M. (2018). La promoción del bienestar en niños hospitalizados a través de una metodología interdisciplinaria. Medicentro Electrónica.

[B26-ejihpe-16-00041] Delamerced A., Panicker C., Monteiro K., Chung E. Y. (2021). Effects of a poetry intervention on emotional wellbeing in hospitalized pediatric patients. Hospital Pediatrics.

[B27-ejihpe-16-00041] Dolidze K., Smith E. L., Tchanturia K. (2013). A model of child life intervention to facilitate effective coping in a child hospitalized for heart surgery. Clinical Practice.

[B28-ejihpe-16-00041] Duda L. J. (2013). Integrating music therapy into pediatric palliative care. Progress in Palliative Care.

[B29-ejihpe-16-00041] Dunlap D., Kagan R. J., Arnold S., Gottschlich M. (2013). “Remember Me” program. Journal of Burn Care & Research.

[B30-ejihpe-16-00041] Ellis S. J., Drew D., Wakefield C. E., Saikal S. L., Punch D., Cohn R. J. (2013). Results of a nurse-led intervention. Journal of Pediatric Oncology Nursing.

[B31-ejihpe-16-00041] Esposito-Smythers C., Spirito A., Kahler C. W., Hunt J., Monti P. (2011). Treatment of co-occurring substance abuse and suicidality among adolescents: A randomized trial. Journal of Consulting and Clinical Psychology.

[B32-ejihpe-16-00041] Fardell J. E., Hu N., Wakefield C., Nassar N., Demetriou E. (2023). Impact of hospitalizations due to chronic health conditions on early child development. Journal of Pediatric Psychology.

[B33-ejihpe-16-00041] Fennig S., Brunstein Klomek A., Shahar B., Sarel-Michnik Z., Hadas A. (2017). Inpatient treatment has no impact on the core thoughts and perceptions in adolescents with anorexia nervosa. Early Intervention in Psychiatry.

[B34-ejihpe-16-00041] Fernández de Córdoba E., Garay M. J. (2018). Leucemia infantil: Necesidades e intervención integral basadas en el modelo centrado en la persona. Educación.

[B35-ejihpe-16-00041] Flujas-Contreras J. M., Ruiz-Castañeda D., Gómez I. (2019). Promoting emotional well-being in hospitalized children and adolescents with virtual reality. CIN Computers Informatics Nursing.

[B36-ejihpe-16-00041] Gallegos S., Álvarez J. C., Gimpel V. E., Bolaño G. F., Toro K. S. (2019). Rol del Terapeuta Ocupacional en tratamiento de niños con delirium hospitalario infantil en unidades hospitalarias de Chile. Revista Chilena de Terapia Ocupacional.

[B37-ejihpe-16-00041] García-Salido A., Calle G. H. L., González A. S. (2018). Revisión narrativa sobre humanización en cuidados intensivos pediátricos: ¿dónde estamos?. Medicina Intensiva.

[B38-ejihpe-16-00041] Gilbey D., Brealey G., Mateo-Arriero I., Waters Z., Ansell M., van Rensburg E. J., Belinelo P. D. G., Milroy H., Pace G., Runions K., Salmin I., Woolard A. (2023). The effectiveness of a day hospital mentalization-based therapy programme for adolescents with borderline personality traits: Findings from touchstone–child and adolescent mental health service. Clinical Psychology & Psychotherapy.

[B39-ejihpe-16-00041] Godino M. J., Martos M. B., Suleiman M., Gómez J. L., Vargas K., Membrive M. J., Albendín L. (2020). Play therapy as an intervention in hospitalized children: A systematic review. Healthcare.

[B40-ejihpe-16-00041] Goldstein G., Mayfield J., Thaler N. S., Walker J., Allen D. N. (2018). Cognitive and academic achievement changes associated with day hospital rehabilitation in children with acquired brain injury. Applied Neuropsychology Child.

[B41-ejihpe-16-00041] González-González C. S., Cáceres-García L., Violant-Holz V. (2019). Bringing computational thinking to hospital classrooms. Proceedings of the seventh international conference on technological ecosystems for enhancing multiculturality.

[B42-ejihpe-16-00041] González-González C. S., Violant Holz V., Infante Moro A., Cáceres García L., Guzmán Franco M. D. (2021). Robótica educativa en contextos inclusivos: El caso de las aulas hospitalarias. Educación XX1.

[B43-ejihpe-16-00041] Gómez Zarco A., Becerra Gálvez A. L., Tron Álvarez R., Hernández Solís P. (2018). Intervención cognitivo-conductual en cuidados paliativos pediátricos: Un caso clínico. Psicooncología.

[B44-ejihpe-16-00041] Grau C. (2005). Educación especial. Orientaciones prácticas.

[B45-ejihpe-16-00041] Hitchcock C., Goodall B., Wright I. M., Boyle A., Johnston D., Dunning D., Gillard J., Griffiths K., Humphrey A., McKinnon A., Panesar I. K., Werner-Seidler A., Watson P., Smith P., Meiser-Stedman R., Dalgleish T. (2022). The early course and treatment of posttraumatic stress disorder in very young children: Diagnostic prevalence and predictors in hospital-attending children and a randomized controlled proof-of-concept trial of trauma-focused cognitive therapy, for 3- to 8-year-olds. Journal of Child Psychology and Psychiatry, and Allied Disciplines.

[B46-ejihpe-16-00041] Hu N., Fardell J., Wakefield C. E., Marshall G. M., Bell J. C., Nassar N., Lingam R. (2022). School academic performance of children hospitalised with a chronic condition. Archives of Disease in Childhood.

[B47-ejihpe-16-00041] Inoue S., Hatakeyama J., Kondo Y., Hifumi T., Sakuramoto H., Kawasaki T., Taito S., Nakamura K., Unoki T., Kawai Y., Kenmotsu Y., Saito M., Yamakawa K., Nishida O. (2019). Post-intensive care syndrome: Its pathophysiology, prevention, and future directions. Acute Medicine & Surgery.

[B48-ejihpe-16-00041] Issaka A., Hopkins L. (2017). Engagement with education: Music education in a paediatric hospital. International Journal of Educational Research.

[B49-ejihpe-16-00041] Kilburn T. R., Sørensen M. J., Thastum M., Rapee R. M., Rask C. U., Arendt K. B., Carlsen A. H., Thomsen P. H. (2023). Group based cognitive behavioural therapy for anxiety in children with autism spectrum disorder: A randomised controlled trial in a general child psychiatric hospital setting. Journal of Autism and Developmental Disorders.

[B50-ejihpe-16-00041] Kompany L., Luis K., Manganaro J., Motacki K., Mustacchio E., Provenzano D. (2016). Children’s specialized hospital and GetWellNetwork collaborate to improve patient education and outcomes using an innovative approach. Pediatric Nursing.

[B51-ejihpe-16-00041] Lagarde C. (2018). A pilot study of the effects of a relaxation movement group on students attending a partial hospitalization program. Doctoral dissertation.

[B52-ejihpe-16-00041] Lee R. (2019). Efficacy of a preparation intervention for the management of children’s pain and fear during needle procedures: Help from a robot named MEDi^®^. Master’s Thesis.

[B53-ejihpe-16-00041] Leyenaar J. K., Schaefer A. P., Freyleue S. D., Austin A. M., Simon T. D., Van Cleave J., Moen E. L., O’Malley A. J., Goodman D. C. (2022). Prevalence of children with medical complexity and associations with health care utilization and in-hospital mortality. JAMA Pediatrics.

[B54-ejihpe-16-00041] Li W., Chung J. O. K., Ho K. Y., Kwok B. M. C. (2016). Play interventions to reduce anxiety and negative emotions in hospitalized children. BMC Pediatrics.

[B55-ejihpe-16-00041] Liszio S., Bäuerlein F., Hildebrand J., van Nahl C., Masuch M., Basu O. (2024). Cooperative virtual reality gaming for anxiety and pain reduction in pediatric patients and their caregivers during painful medical procedures: Research protocol for a randomized control trial (Preprint). JMIR Research Protocols.

[B56-ejihpe-16-00041] Lizasoain O. (2021). De qué hablamos cuando hablamos de pedagogía hospitalaria. Edutec Revista Electrónica de Tecnología Educativa.

[B57-ejihpe-16-00041] Logan D. E., Breazeal C., Goodwin M. S., Jeong S., O’Connell B., Smith-Freedman B., Heathers J., Weinstock P. (2019). Social robots for hospitalized children. Pediatrics.

[B58-ejihpe-16-00041] Major J., Varga Z. K., Gyimesi-Szikszai A., Ádám S. (2017). A two-week inpatient programme with a booster improved long-term management of severe chronic paediatric pain. Journal of Child Health Care.

[B59-ejihpe-16-00041] McClure V. (2017). The impact of the Microsoft Office PowerPoint program on students with medical conditions in a home and hospital program. Doctoral dissertation.

[B60-ejihpe-16-00041] Meentken M. G., van der Ende J., del Canho R., van Beynum I. M., Aendekerk E. W. C., Legerstee J. S., Lindauer R., Hillegers M. H. J., Helbing W. A., Moll H. A., Utens E. M. W. J. (2021). Psychological outcomes after pediatric hospitalization: The role of trauma type. Children s Health Care.

[B61-ejihpe-16-00041] Munn Z., Peters M. D. J., Stern C., Tufanaru C., McArthur A., Aromataris E. (2018). Systematic review or scoping review? Guidance on selecting the review approach for evidence synthesis. BMC Medical Research Methodology.

[B62-ejihpe-16-00041] Muñoz-Arteaga J., Amador C. V., Reyes H. C., Esparza M. O., Aguiñaga C. O. (2021). Process model to develop educational applications for hospital school programs. New perspectives in software engineering.

[B63-ejihpe-16-00041] National Academies of Sciences, Engineering, and Medicine [NASEM] (2024). Launching lifelong health by improving health care for children, youth, and families.

[B64-ejihpe-16-00041] Nguyen T. N., Nilsson S., Hellström A. L., Bengtson A. (2010). Music therapy to reduce pain and anxiety in children with cancer undergoing lumbar puncture: A randomized clinical trial. Journal of Pediatric Oncology Nursing.

[B65-ejihpe-16-00041] Noblecilla Castro R. M. (2019). Recursos digitales para mejorar el desarrollo académico y emocional de niños y adolescentes hospitalizados en el Instituto Nacional de Salud del Niño. Master’s thesis.

[B66-ejihpe-16-00041] Olson K., Sands S. A. (2015). Cognitive training programs for childhood cancer patients and survivors: A critical review and future directions. Child Neuropsychology.

[B67-ejihpe-16-00041] Pan American Health Organization (2024). Child health.

[B68-ejihpe-16-00041] Payam S., Hossaini J., Zaschka K., Friedmann A., Mall V. (2023). Designing well-being: A qualitative investigation of young patients’ perspectives on the material hospital environment. HERD Health Environments Research & Design Journal.

[B70-ejihpe-16-00041] Peters M. D. J., Godfrey C., McInerney P., Munn Z., Tricco A. C., Khalil H., Aromataris E., Lockwood C., Porritt K., Pilla B., Jordan Z. (2024). Chapter 11: Scoping reviews. JBI manual for evidence synthesis.

[B69-ejihpe-16-00041] Peters M. D. J., Godfrey C. M., Khalil H., McInerney P., Parker D., Soares C. B. (2015). Guidance for conducting systematic scoping reviews. International Journal of Evidence-Based Healthcare.

[B71-ejihpe-16-00041] Pollard E. L., Lee P. D. (2003). Child well-being: A systematic review of the literature. Social Indicators Research.

[B72-ejihpe-16-00041] Potasz C., De Varela M. J., De Carvalho L. C., Do Prado L. F., Do Prado G. F. (2013). Effect of play activities on hospitalized children’s stress: A randomized clinical trial. Scandinavian Journal of Occupational Therapy.

[B73-ejihpe-16-00041] Pottinger H. L. (2017). Integrative wellness sessions in a pediatric hospital setting: A mixed-methods feasibility study to assess evaluation of the Hospital Heroes program at Banner’s Diamond Children’s Hospital.

[B74-ejihpe-16-00041] Ramachandran S., Stensland S., Corder J. H., Cuomo C., Dao T.-V., Natowicz M. R. (2025). Adolescent to adult health care transition for persons with intellectual and developmental disability: Current barriers, next steps. Frontiers in Pediatrics.

[B75-ejihpe-16-00041] Ranamagar B., Karki S. (2021). The effectiveness of handicrafts on anxiety reduction among hospitalised children in paediatric ward of dhulikhel hospital. Journal of Nepal Paediatric Society.

[B76-ejihpe-16-00041] Reif L. K., van Olmen J., McNairy M. L., Ahmed S., Putta N., Bermejo R., Nugent R., Paintsil E., Daelmans B., Varghese C., Sugandhi N., Abrams E. J. (2022). Models of lifelong care for children and adolescents with chronic conditions in low-income and middle-income countries: A scoping review. BMJ Global Health.

[B77-ejihpe-16-00041] Reitze A., Voigt M., Klawonn F., Dusch M., Grigull L., Mücke U. (2024). Impact of virtual reality on peri-interventional pain, anxiety and distress in a pediatric oncology outpatient clinic: A randomized controlled trial. BMC Pediatrics.

[B78-ejihpe-16-00041] Ridout B., Kelson J., Campbell A., Steinbeck K. (2021). Effectiveness of virtual reality interventions for adolescent patients in hospital settings: Systematic review. Journal of Medical Internet Research.

[B79-ejihpe-16-00041] Rosenblatt A., Pederson R., Davis-Sandfoss T., Irwin L., Mitsos R., Manworren R. (2024). Child life specialist services, practice, and utilization across health care: A scoping review. JBI Evidence Synthesis.

[B80-ejihpe-16-00041] Ryan R. M., Deci E. L. (2001). On happiness and human potentials: A review of research on hedonic and eudaimonic well-being. Annual Review of Psychology.

[B81-ejihpe-16-00041] Ryff C. D. (1989). Happiness is everything, or is it? Explorations on the meaning of psychological well-being. Journal of Personality and Social Psychology.

[B82-ejihpe-16-00041] Ryff C. D., Keyes C. L. M. (1995). The structure of psychological well-being revisited. Journal of Personality and Social Psychology.

[B83-ejihpe-16-00041] Sánchez J. C., Porras G. L., Viera M. T., Olaya J. C., García A., Muñoz L. V., Mesa H., Ramírez A. F. (2024). Effects of clowning on anxiety, stress, pain, and hormonal markers in paediatric patients. BMC Pediatrics.

[B84-ejihpe-16-00041] Sil S., Lee J. L., Klosky J., Vaz A., Mee L., Cochran S., Thompson B., Coakley R. (2021). The comfort ability program for adolescents with sickle cell pain: Evaluating feasibility and acceptability of an inpatient group-based clinical implementation. Pediatric Blood & Cancer.

[B85-ejihpe-16-00041] Sillero Sillero A., Ayuso Margañon R., Marques-Sule E., Gil Poisa M. (2024). Child-centered care: A qualitative study exploring pediatric hospitalization through children’s perspectives. Nursing Reports.

[B86-ejihpe-16-00041] Spencer B. K. C., Wright J., Flemming K., Cottrell D., Pini S. (2023). School lives of adolescent school students living with chronic physical health conditions: A qualitative evidence synthesis. Archives of Disease in Childhood.

[B87-ejihpe-16-00041] Stadler H., Müller K., Kurlemann G., Lendt M. (2024). Effectiveness of neuropediatric inpatient rehabilitation. Neuropediatrics.

[B88-ejihpe-16-00041] Suleman S. K., Atrushi A., Enskär K. (2022). Effectiveness of art-based distraction in reducing pain and anxiety of hospitalized children during cannulation procedure: A randomized controlled trial. Belitung Nursing Journal.

[B89-ejihpe-16-00041] Taguchi K., Ueno T., Shimizu Y., Ishimoto R., Hada Y. (2018). Effect of inpatient rehabilitation on activities of daily living in pediatric cancer patients in Japan. International Journal of Rehabilitation Research.

[B90-ejihpe-16-00041] Thabrew H., Chubb L. A., Kumar H., Fouché C. (2022). Immersive reality experience technology for reducing social isolation and improving social connectedness and well-being of children and young people who are hospitalized: Open trial. JMIR Pediatrics and Parenting.

[B91-ejihpe-16-00041] Toyama H. (2021). Graded exposure treatment (GET) for pediatric chronic pain and its effect on parent-child dyads: The pain is relative. Doctoral dissertation.

[B92-ejihpe-16-00041] Tricco A. C., Lillie E., Zarin W., O’Brien K. K., Colquhoun H., Levac D., Moher D., Peters M. D. J., Horsley T., Weeks L., Hempel S., Akl E. A., Chang C., McGowan J., Stewart L., Hartling L., Aldcroft A., Wilson M. G., Garritty C., Straus S. E. (2018). PRISMA extension for scoping reviews (PRISMA-ScR): Checklist and explanation. Annals of Internal Medicine.

[B93-ejihpe-16-00041] United Nations (1989). Convention on the rights of the child. *Office of the High Commissioner for Human Rights*.

[B94-ejihpe-16-00041] United Nations (2024). UN population division data portal. Interactive access to global demographic indicators. Life expectancy at birth.

[B95-ejihpe-16-00041] Veritas Health Innovation (2024). Covidence systematic review software.

[B96-ejihpe-16-00041] Villadsen K. W., Blix C., Boisen K. A. (2015). More than a break: The impact of a social-pedagogical intervention during young persons’ long-term hospital admission—A qualitative study. International Journal of Adolescent Medicine and Health.

[B97-ejihpe-16-00041] Viotti F. (2018). La calidad de vida relacionada con la salud y el uso del tiempo en niños hospitalizados. Doctoral thesis.

[B98-ejihpe-16-00041] Wallander J. L., Varni J. W. (1998). Effects of pediatric chronic physical disorders on child and family adjustment. Journal of Child Psychology and Psychiatry.

[B99-ejihpe-16-00041] World Health Organization (2016). Framework on integrated, people-centred health services: Report by the director-general.

[B100-ejihpe-16-00041] World Health Organization (2025). Child deaths: Under-five mortality rate (per 1000 live births) (SDG 3.2.1).

[B101-ejihpe-16-00041] Zaccagnini M., Li J. (2023). How to conduct a systematic review and meta-analysis: A guide for clinicians. Respiratory Care.

[B102-ejihpe-16-00041] Zamani M., Sigaroudi A. E., Pouralizadeh M., Kazemnejad-Leili E. (2022). Effect of the Digital Education Package (DEP) on prevention of anxiety in hospitalized children: A quasi-experimental study. BMC Nursing.

[B103-ejihpe-16-00041] Zucchetti G., Ciappina S., Geuna T., Nichelli F., Biondi A., Camera F., Ripaldi M., Fagioli F. (2020). Effects of hospital early childcare intervention in young children with cancer. Child Care in Practice.

